# Definition of treatment-resistant late-life depression: Conclusions from a European Task Force Delphi process

**DOI:** 10.1192/j.eurpsy.2026.12228

**Published:** 2026-06-02

**Authors:** B. Pozuelo Moyano, A. von Gunten, C. Mueller, R. Yaman-Deveci, R. Howard, R. Stewart, A.H. Young, H. Costello, S. Bulteau, B. Calvet, S. Bonin-Guillaume, N. Hoertel, G.-H. Robert, J. Roche, A. Lepetit, S. Louchart de la Chapelle, L.F. Agüera-Ortiz, J. Pla-Vidal, F. Bouckaert, R.C. Oude Voshaar, L. Fernandes, O. Vasiliu, M. Dominiak, J. Priller, M. Preisig, F. Triolo, M. Belvederi Murri, E. Aakhus, S. Ranjbar, K. Swierkosz-Lenart, V. Orgeta, P. Vandel

**Affiliations:** 1 Service of Old Age Psychiatry, Department of Psychiatry, Lausanne University Hospital (CHUV) and University of Lausanne (UNIL), Prilly, Switzerland; 2 Department of Psychological Medicine, Institute of Psychiatry, Psychology and Neuroscience (IoPPN), King’s College London, London, United Kingdom; 3 South London and Maudsley NHS Foundation Trust, London, UK; 4 Division of Psychiatry, Faculty of Brain Sciences, University College London (UCL), London, United Kingdom; 5 Division of Psychiatry, Department of Brain Sciences, Imperial College London, London, United Kingdom; 6 CHU Nantes, department of Old Age Psychiatry, Nantes, France; 7 UMR INSERM 1246, SPHERE, University of Nantes and University of Tours, Nantes, France; 8 Inserm U1094, IRD UMR270, Univ. Limoges, CHU Limoges, EpiMaCT – Epidemiology of Chronic Diseases in Tropical Zone, Institute of Epidemiology and Tropical Neurology, OmegaHealth, Limoges, France; 9 Centre Jean-Marie Léger, Pôle Universitaire de Psychiatrie de l’Adulte et de la Personne Âgée, d’Addictologie (PUP3A), CH Esquirol, Limoges, France; 10 Assistance Publique des Hôpitaux de Marseille, UMR INSERM 1106, Aix-Marseille Université, Marseille, France; 11 AP-HP.Centre, DMU Psychiatrie et Addictologie, Corentin Celton Hospital, Issy-les-Moulineaux, France; 12 INSERM UMR_1266, Institut de Psychiatrie et Neuroscience de Paris, Paris, France; 13 Université Paris Cité, Faculté de Santé, UFR de Médecine, Paris, France; 14 Pôle Hospitalo-Universitaire de Psychiatrie Adulte, Centre Hospitalier Guillaume Régnier, 108 Bd Général Leclerc, 35000, Rennes, France; 15 Empen, U1288, IRISA UMR 6074, Campus Beaulieu, Université de Rennes, France; 16 Service of Old Age Psychiatry, Department of Geriatry, University Hospital of Lille, Lille, France; 17 Regional Centre of Psychogeriatry and Old Age Psychiatry (CR3PA), Lille, France; 18 Hospices Civils de Lyon, Hôpital des Charpennes, Villeurbanne, France; 19 Université Claude Bernard Lyon 1, CNRS, INSERM, Centre de Recherche en Neurosciences de Lyon CRNL U1028 UMR5292, F-69500, Bron, France; 20 RAINIER III Clinical Gerontology Center, Memory Clinic and Clinical Research Unit, Princess Grace Hospital, Monaco; 21 Department of Psychiatry, Instituto de Investigación Sanitaria (imas12), Hospital Universitario 12 de Octubre, & CIBERSAM, Instituto de Salud Carlos III, Madrid, Spain; 22 Department of Psychiatry and Medical Psychology, Clínica Universidad de Navarra, Madrid, Spain; 23 KU Leuven, Leuven Brain Institute, Department of Neurosciences, Neuropsychiatry, Leuven, Belgium; 24 Geriatric Psychiatry, University Psychiatric Center KU Leuven, Leuven, Belgium; 25 Department of Psychiatry, University of Groningen, University Medical Center Groningen (UMCG), Groningen, The Netherlands; 26 RISE-Health, Department of Clinical Neuroscience and Mental Health, Faculty of Medicine, University of Porto, Porto, Portugal; 27 Psychiatry Service, Unidade Local de Saúde (ULS) São João, Porto, Portugal; 28 Discipline of Psychiatry, Clinical Neurosciences Department, Carol Davila University of Medicine and Pharmacy, Bucharest, Romania; 29 Institute of Psychiatry and Neurology, ul. Sobieskiego 9, 02-957 Warsaw, Poland; 30 Neuropsychiatry and Laboratory of Molecular Psychiatry, Charité – Universitätsmedizin Berlin, and German Center for Neurodegenerative Diseases (DZNE), Berlin, Germany; 31 Department of Psychiatry and Psychotherapy and German Center for Mental Health (DZPG), School of Medicine and Health, Technical University of Munich, Munich, Germany; 32 University of Edinburgh and UK Dementia Research Institute, Edinburgh, United Kingdom; 33 Psychiatric Epidemiology and Psychopathology Research Center, Department of Psychiatry, Lausanne University Hospital and University of Lausanne, Prilly, Switzerland; 34 Aging Research Center, Department of Neurobiology, Care Sciences and Society, Karolinska Institutet and Stockholm University, Stockholm, Sweden; 35 Department of Psychiatry, Amsterdam UMC, Vrije Universiteit Amsterdam, Amsterdam, The Netherlands; 36 Institute of Psychiatry, Department of Neuroscience and Rehabilitation, University of Ferrara, Ferrara, Italy; 37 Integrated Department of Mental Health and Pathological Addictions, Local Health Trust of Ferrara, Ferrara, Italy; 38 Norwegian National Centre for Ageing and Health, Vestfold Hospital Trust, Tønsberg, Norway; 39 Department of Psychiatry, Center for Research in Psychiatric Epidemiology and Psychopathology, Lausanne University Hospital, University of Lausanne, Prilly, Switzerland

**Keywords:** definitional criteria, European consensus, TRLLD

## Abstract

**Background:**

Treatment-Resistant Late-Life Depression (TRLLD) represents a significant clinical and research challenge. Studies on TRLLD use heterogeneous diagnostic criteria limiting the interpretation of results and the development of clinical guidelines.

This study aimed to establish expert consensus on the core components and definition of TRLLD through a Delphi process conducted by a European Task Force of clinicians and researchers with experience in late-life depression.

**Methods:**

We conducted an electronic Delphi study with 30 European experts to identify key definitional elements of TRLLD. In the 1st survey round (SR), participants responded to open-ended questions on definitions of TRLLD. Responses informed the development of 70 structured items for 2nd SR, involving 58 closed (yes/no) items and 12 multiple-choice questions across six domains: symptom presentation, cognitive impairment, comorbidities, pharmacotherapy, treatment adherence, and psychosocial factors. Consensus was defined as ≥70% agreement. In 3rd SR, participants reviewed comments and validated the final categorical definition, the operational staging model, and the derived decision algorithm for TRLLD.

**Results:**

Consensus was reached for 72.4% of the 58 closed items. Experts agreed that the categorical definition of TRLLD should correspond to major depressive disorder in individuals aged ≥65 years who show insufficient response to two adequate antidepressant treatments, in the absence of dementia and medical conditions that could account for depressive symptoms. An operational staging model and decision-making algorithm were developed from consensus items.

**Conclusion:**

This study provides the first European consensus definition of TRLLD and highlights the need for age-adapted diagnostic criteria that reflect the clinical complexity of depression in older adults.

## Introduction

Depression is common among older adults, with a 12-month prevalence of around 5% for DSM-5 major depressive disorder (MDD) among individuals aged 65 and older [1]. It is a leading cause of disability and preventable morbidity worldwide [2–5]. Despite advances in treatment options for late-life depression (LLD), response rates are still strikingly low: only about half of older adults achieve a meaningful response to antidepressant medications [6].

Treatment-resistant late-life depression (TRLLD) is a leading reason for consultation in psychiatry, geriatric medicine, and primary care [7]. It causes a substantial healthcare and economic burden [7, 8] and is associated with increased mortality, including suicide, compared with non-resistant LLD patients [9]. TRLLD often co-occurs with multiple somatic diseases and has been linked to incident dementia [9–11]. With the rapid ageing of the European population [12], extending the knowledge of clinical features and management of TRLLD is a public health priority.

Current research is hindered by the absence of a consistent framework regarding its diagnostic definition, which limits comparability across studies and reduces the applicability of clinical evidence [9, 13–17]. This heterogeneity is reflected in randomised clinical trials (RCTs): among TRLLD RCTs conducted to date, approximately half define resistance as non-response to a single antidepressant trial, while the other half define it as non-response to two treatments, making the overall results difficult to interpret [14].

One of the operational staging models commonly used to define treatment-resistant depression is the Thase and Rush model (1997), which grades resistance according to the number and type of failed treatment steps, but was originally developed for the general adult population, rather than being specific to LLD [18]. More recently, Patrick et al. (American NNDC Geriatric Mood Disorders Task Group, 2024) proposed a definition of TRLLD based on the presence of MDD in adults aged ≥60 years (with age of onset specified), where MDD is the primary diagnosis (i.e., not secondary to dementia or another medical condition) and there is inadequate response to ≥2 adequate antidepressant trials or to a course of ECT or rTMS, with treatment adequacy judged against accepted dose and duration standards [9]. However, substantial variability in expert opinion persists regarding core components of TRLLD, including how to evaluate adherence to pharmacological treatment, how to account for the high burden of physical health comorbidities, and which treatment modalities – pharmacological and non-pharmacological – should be considered when determining resistance.

Given the absence of consensus and the multidimensional nature of TRLLD, a Delphi process is particularly suitable for integrating expert judgment. This position paper reports the findings of a Delphi study conducted by a European panel of clinicians and researchers with extensive experience in TRLLD. The aim was to systematically assess the current views and establish consensus on the essential definitional criteria of TRLLD.

## Methods

### Study design

We conducted an electronic Delphi (e-Delphi) study to systematically synthesise expert opinions and reach an informed consensus on the resistance criteria for depression in older people [19].

### Sample

#### Steering committee

The steering committee for this e-Delphi study was based in Switzerland and the UK, and comprised four members: three clinical academic old-age psychiatrists (BPM, AvG, and PV), and one academic psychologist (VO). The committee was responsible for overseeing the design of the study. Monthly meetings were held to maintain study rigour and manage data collection.

#### Selection of experts

Clear selection criteria were applied to minimise researcher bias based on the procedure outlined by Gill et al. [20] (Table 1).

**Table 1.** Expert selection criteria and implementation, based on the procedure outlined by Gill et al. [20] Table 1. long description.

Selection criteria	How it was implemented in this study
Identifying the most appropriate categories of experts for the panel	We identified experts in old-age psychiatry, geriatrics, psychology, neurology, and related fields with expertise in LLD. The experts were selected for clinical or research experience in old-age psychiatry and/or geriatrics. Our recruitment strategy aimed to achieve broad European geographical representation, thereby enriching the panel with diverse perspectives on TRLLD.
Identifying experts	Names were compiled from various sources, including previous research participation [14], publications on LLD or TRLLD, and membership of national or European professional associations or societies. Most expert panellists were independent academic clinicians and reported no relevant conflicts of interest. The main coordination of the Delphi study was undertaken by a steering committee comprised of independent researchers.
Contacting individuals	Experts were contacted and invited to nominate additional professionals whom they believed could provide valuable insights. This snowball sampling approach was used to ensure a broad and diverse pool of expertise.
Creating sub-lists for each stakeholder group	Experts were grouped according to professional background, including psychology, geriatrics, psychiatry, neurology, and old-age psychiatry.
Inviting the experts	We aimed to assemble a convenience sample of at least 30 experts.

### Recruitment procedure

We emailed invitation letters to 30 experts from 14 European countries. Interested parties could access the questionnaire via a direct URL link.

### Survey instrument and data collection procedures

The tools Google Forms (for the 1st SR) and SurveyMonkey® (for the 2nd SR) were employed. Each e-Delphi SR remained open for a minimum of 4 weeks [20].

### Delphi survey rounds

**Figure 1.**
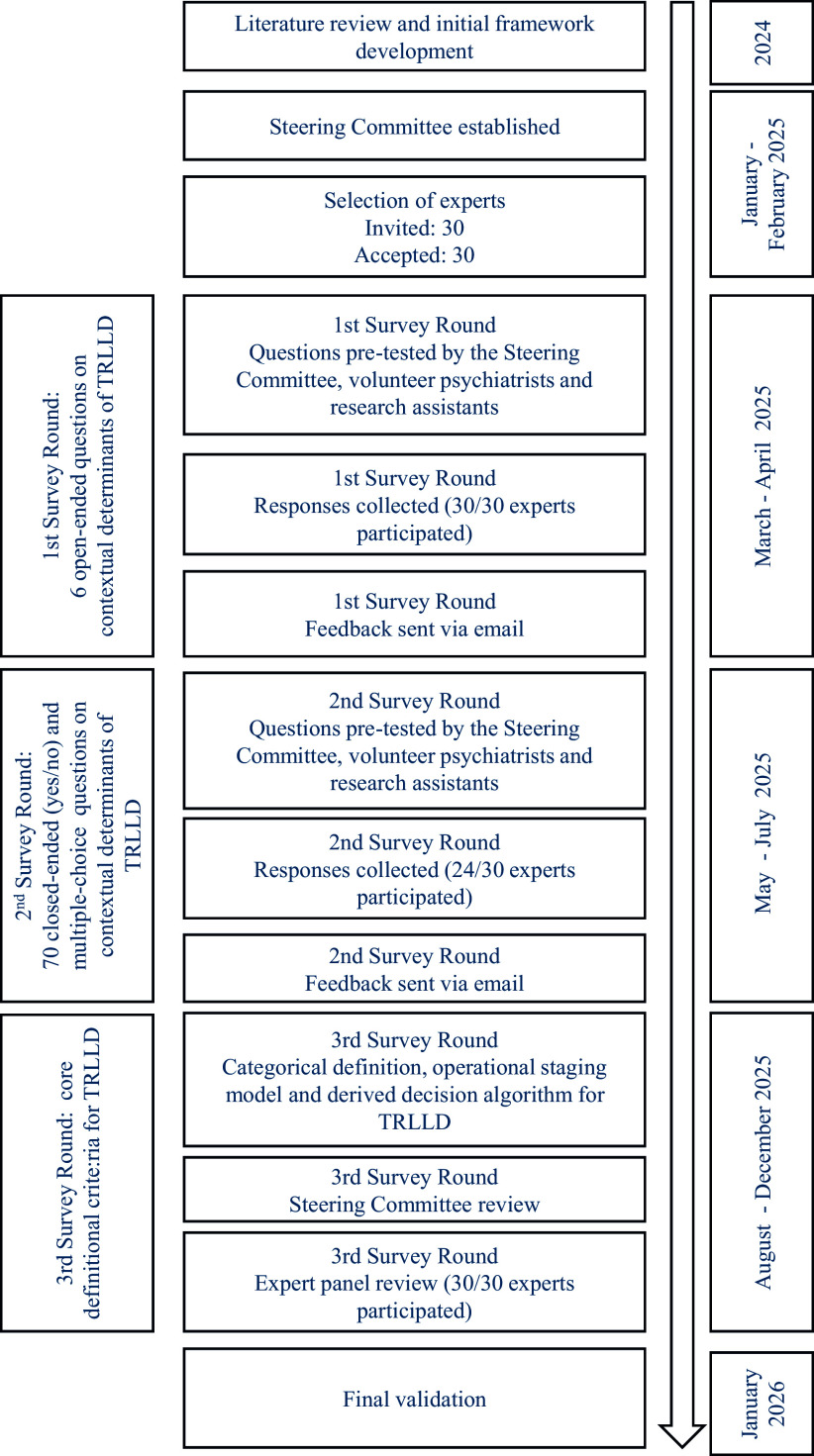
Delphi method flowchart. Figure 1. long description.

We started with a 1st SR using open questions, followed by a 2nd SR with a high proportion of closed questions, including contextual determinants relevant to the definition of TRLLD. For the 2nd SR, the consensus threshold was defined a priori, specifying that a minimum of 70% agreement would be required to consider an item as having reached consensus. Delphi methodology does not prescribe a single universally required level of agreement, and consensus thresholds should be predefined and justified according to the study’s aims [21]. We selected a ≥70% threshold, consistent with previous psychiatric Delphi research on depression-related topics, in which similar agreement thresholds have been used to define or support consensus [22, 23]. We concluded with a 3rd SR, in which all Delphi panel members were invited to review and validate the final core definitional criteria for TRLLD (Figure 1). An explanatory email was sent to participants before each SR.

### Measures

In order to capture the current criteria and heterogeneity in practice, as well as global perspectives (Table 2), in the 1st SR, the expert panel responded to six open questions (Table 3), focusing on defining contextual determinants relevant to the definition of TRLLD [24]. These responses informed the development of 70 questions for the 2^nd^ SR, comprising 58 closed questions (yes/no or relevant/not relevant) and 12 open or multiple-choice questions. These questions aimed to refine and consolidate the previously gathered perspectives. The 70 items were organised into six thematic sections: (1) global definition and clinical presentation, (2) co-occurrence of cognitive impairment, dementia, and vascular depression, (3) co-occurrence of other physical and mental comorbidities, (4) pharmacokinetics, pharmacodynamics, and drug interactions, (5) treatment adherence and tolerance, and (6) social and psychological factors. Each section included a final free-text comment field. Four additional demographic questions were included. The questions were pretested by the steering committee and additional volunteer psychiatrists and research assistants at Lausanne University Hospital. Consensus was defined as the agreement of at least 70% in the closed questions [21].

**Table 2.** Demographics and professional background of the Delphi participants Table 2. long description.

Item	1st SR (*n* = 30)	2nd SR (*n* = 24)	Experts not completing 2nd SR (*n* = 6)
Primary professional background			
Psychiatry (general)	16 (53.3%)	13 (54.2%)	3 (50.0%)
Old age psychiatry*	10 (33.3%)	7 (29.2%)	3 (50.0%)
Geriatrics	2 (6.7%)	2 (8.3%)	0 (0.0%)
Neurology*	1 (3.3%)	1 (4.2%)	0 (0.0%)
Psychology	1 (3.3%)	1 (4.2%)	0 (0.0%)
Years of professional experience			
6–9 years	3 (10.0%)	3 (12.5%)	0 (0.0%)
10–20 years	12 (40.0%)	12 (50.0%)	0 (0.0%)
>20 years	15 (50.0%)	9 (37.5%)	6 (100.0%)
Clinical degree/academic background			
MD (no PhD)	11 (36.7%)	9 (37.5%)	2 (33.3%)
PhD (with or without MD)	19 (63.3%)	15 (62.5%)	4 (66.7%)
Country of participants			
Belgium	1 (3.3%)	1 (4.2%)	0 (0.0%)
England	6 (20.0%)	4 (16.7%)	2 (33.3%)
France	7 (23.3%)	5 (20.8%)	2 (33.3%)
Germany	1 (3.3%)	1 (4.2%)	0 (0.0%)
Italy	1 (3.3%)	1 (4.2%)	0 (0.0%)
Monaco	1 (3.3%)	0 (0.0%)	1 (16.7%)
Netherlands	1 (3.3%)	1 (4.2%)	0 (0.0%)
Norway	1 (3.3%)	1 (4.2%)	0 (0.0%)
Poland	1 (3.3%)	1 (4.2%)	0 (0.0%)
Portugal	1 (3.3%)	1 (4.2%)	0 (0.0%)
Romania	1 (3.3%)	1 (4.2%)	0 (0.0%)
Spain	2 (6.7%)	2 (8.3%)	0 (0.0%)
Sweden	1 (3.3%)	1 (4.2%)	0 (0.0%)
Switzerland	5 (16.7%)	4 (16.7%)	1 (16.7%)

* Some participants reported dual specialties (e.g., Psychiatry & Geriatrics; Psychiatry & Neurology). For clarity, these were grouped under the most relevant primary category (Old age psychiatry or Neurology, respectively). Psychology participant was PhD (no MD).

**Table 3.** 1st SR open-ended questions Table 3. long description.

Question
How would you define treatment-resistant late-life depression (TRLLD)?
How many ineffective treatment trials should be required to define treatment resistance?
Which classes of treatments should be considered when evaluating an unsuccessful attempt to treat late-life depression?
Do you think unsuccessful psychological treatments should be included when defining TRLLD?
Should the biological causes and mechanisms of disease investigations be included when defining TRLLD?
Are there any criteria for defining treatment-resistant depression that should be specifically considered in the older population compared with the adult population?

The materials provided to the panel were primarily informed by a recent systematic review conducted by some members of the Task Force (covering the literature up to March 2024) [14]. The study was conducted in accordance with the Conducting and Reporting Delphi Studies (CREDES) guidelines (Supplementary Table S1) [25], thereby ensuring methodological transparency and replicability.

### Participant feedback

At the end of the 1st SR and 2nd SR, all panellists received an anonymised summary of aggregate results (item-level agreement percentages) and synthesised qualitative comments from the free-text fields. At the end of the 3rd SR, participants received feedback on their comments regarding the categorical definition, the operational staging model, and the decision algorithm, either through comments in the manuscript or via email.

## Results

The study was conducted from January 2025 to January 2026. The characteristics of the panel in terms of medical specialty, professional experience, country of practice, and academic background in the 1st and 2nd SR, as well as those of the non-completers of the 2nd SR, are presented in Table 2.

### First survey round

#### First survey round findings

All panel members completed the 1st SR questions. Overall, 70% of the panel recommended that two unsuccessful antidepressant trials were necessary to define TRLLD. Participants were then asked whether unsuccessful psychological treatments should be included in defining treatment resistance; 37% supported the inclusion of this. Additionally, 57% of participants emphasised the importance of considering the biological causes and mechanisms of the disease in this context.

#### Late-life specific considerations informing the second survey round

At the end of the 1st SR, participants were invited to propose criteria for defining treatment-resistant depression that should be specifically considered in the older population. A total of 13 experts highlighted comorbidities and medical complexity, and 13 focused on cognitive impairment and dementia-related comorbidities. Eight participants referenced pharmacokinetic, pharmacodynamic, and dosing challenges. Six noted the atypical symptom presentation and diagnostic difficulties in this population. Six proposed adopting criteria based on existing adult guidelines. Four emphasised polypharmacy and drug interactions, four cited treatment adherence and tolerance, and four identified social and psychological factors as important considerations. These insights served as the foundation for constructing the 2nd SR of structured questions on contextual determinants relevant to the definition of TRLLD.

### Second survey round

A total of 80% (24/30) of participants completed the 2nd SR. Consensus was achieved for 72.4% of the closed questions. Tables 4 and 5 present the level of agreement across the various questions in each section. Table 6 summarises the responses to the multiple-choice questions. Additionally, the free-text field included the following comments from members, organized by section as detailed below (Table 7).

**Table 4.** Consensus-endorsed statements from the 2^nd^ SR identifying contextual determinants relevant to the definition of TRLLD Table 4. long description.

Category 1: Global definition and clinical presentation*Age definition & diagnostic criteria*	
Age-specific TRLLD criteria should be developed to better reflect the needs of older populations.	92%
It is important to define TRLLD as applying to individuals aged 65 years and older.*	79%
Individuals with the onset of treatment-resistant depression (TRD) at age 85 or older (≥85) should not be excluded from the definition of TRLLD.*	83%
There should be a minimum severity threshold for diagnosing TRLLD.*	75%
The minimum severity threshold for diagnosing TRLLD should be moderate depressive episode.	90%
The definition of TRLLD should specify whether it refers to early-onset or late-onset TRLLD.	96%
The distinction between recurrent depressive disorder and a first episode LLD should be specified in the definition of TRLLD.*	83%
TRLLD should include cases where older adults with a history of recurrent depressive disorder develop resistance to an antidepressant that was previously effective.	88%

Category 1: Global definition and clinical presentation*Operational versus categorical definitions of TRLLD*	
TRLLD should be defined operationally – rather than as a fixed category – by using a continuum or staging model based on the number and type of failed treatment attempts [18].*	83%
Existing TRD staging models [18] should be adapted for older adults. *	83%
If TRLLD should be defined operationally, the following TRLLD model should be used: Stage II: Failure of two adequate trials from two distinct classes. *	78%
If TRLLD should be defined operationally, the following TRLLD model should be used: Stage III: Stage II plus failure of augmentation (e.g., aripiprazole, lithium, methylphenidate). *	83%
If TRLLD should be defined operationally, the following TRLLD model should be used: Stage IV: Stage III plus failure of Electroconvulsive Therapy (ECT), repetitive Transcranial Magnetic Stimulation (rTMS), or ketamine.*	71%
In research, TRLLD should also be defined operationally rather than as a fixed category.	88%
If TRLLD should be defined operationally, the model should not begin with psychotherapy at Stage I.*	75%

Category 1: Global definition and clinical presentation*Number of treatment failures & psychotherapy*	
TRLLD definition should include a failure to respond to two adequate antidepressant trials.*	83%
Psychotherapeutic interventions should not be counted as one failed treatment when defining TRLLD.*	71%
Psychotherapy interventions should be defined as one out of three failed treatments used to define TRLLD.*	82%

Category 1: Global definition and clinical presentation*Treatment duration and evaluation timeline*	
A specific minimum duration for an antidepressant trial in older adults is necessary to adequately assess treatment response in LLD.	100%
Although the literature suggests that antidepressant response in older patients may require 10–12 weeks, this duration is considered too long for the demands of real-world clinical practice.	88%
Clinicians should follow the principle “start low, go slow, but go,” using, if needed, the same minimum effective doses as in younger adults unless contraindicated.	92%

Category 1: Global definition and clinical presentation*Symptom presentation in older adults*	
The diagnostic criteria for TRLLD should include atypical and masked symptoms commonly observed in older adults.	92%
The differences between improvements observed by clinicians and reported by patients should be considered when defining TRLLD.	96%
Discrepancies between symptom reduction and functional improvement should be considered in the TRLLD definition.	96%

Category 1: Global definition and clinical presentation*Assessment tools*	
The Montgomery–Åsberg Depression Rating Scale (MADRS) is an appropriate tool for monitoring symptoms in TRLLD.	88%
The Hamilton Depression Rating Scale (HAM-D) is an appropriate tool for monitoring symptoms in TRLLD.	88%
The Cornell Scale for Depression in Dementia is an appropriate tool for monitoring symptoms in TRLLD.*	79%

Category 2: Cognitive impairment, dementia, vascular depression	
Cognitive impairment can interfere with the accurate diagnosis of TRLLD.	96%
Cognitive impairment can affect treatment response in LLD.	100%
Cognitive screening should be routinely included in the TRLLD assessment.	92%
Older adults with TRD, no prior history of depressive episodes, and dementia should not be included in the definition of TRLLD. They should be classified separately (e.g., as behavioural and psychological symptoms of dementia with treatment-resistant depressive symptoms).*	75%
In patients with LLD, if apathy is the only remaining symptom after two or three adequate treatment trials, this does not indicate a TRLLD.*	75%

Category 3: Physical or mental comorbidities and TRLLD	
Certain conditions (e.g., thyroid disorders, testosterone deficiency, anaemia, vitamin deficiencies, etc.) should be screened for before diagnosing TRLLD.	92%
If a patient has persistent depressive symptoms in the context of another primary psychiatric disorder despite receiving adequate treatment, this does not indicate TRLLD.*	83%
Physical comorbidities (e.g., chronic pain or hypertension) should be adequately managed before confirming a diagnosis of TRLLD.*	79%

Category 4: Pharmacokinetics, pharmacodynamics, drug interactions and TRLLD	
Age-specific pharmacokinetic and pharmacodynamic characteristics should be considered when defining TRLLD.	96%

Category 5: Treatment adherence, tolerance and TRLLD	
Poor adherence is a common cause of treatment failure in older adults with depression.	96%
In patients with Late-Life Depression (LLD), if one pharmacological treatment was discontinued due to side effects, it should not be considered a failed trial, even if depressive symptoms improved with this treatment.	92%
The standard TRLLD assessment should include structured questions about adherence, with input from a proxy such as a partner or caregiver.*	83%
Older adults with cognitive impairment require additional adherence monitoring before being classified as TRLLD.	88%

Category 6: Social, psychological factors and TRLLD	
Social isolation contributes to poor treatment outcomes in LLD.	92%
Psychosocial stressors (e.g., financial difficulties, recent losses, or institutional living) should be routinely assessed before diagnosing TRLLD.*	83%

*Note*: Percentages indicate the proportion of participating experts in the 2nd SR (*n* = 24) who endorsed each statement. Items marked with one asterisk (*) fell below the 70% consensus threshold under the worst-case scenario, assuming that all six experts who did not complete the 2nd SR would have disagreed. See Supplementary Table S2 for the full sensitivity analysis.

**Table 5.** Non-consensus statements in the 2nd SR Table 5. long description.

Category 1: Global definition and clinical presentation*Age definition and diagnostic criteria*	
Current adult TRD criteria are generally applicable to older adults.	62%
Adults aged ≥85 years should be considered a distinct diagnostic subgroup (e.g., Very Late-Onset-TRD).	58%
In recurrent depression, if a previously effective antidepressant no longer works, it should not count as an adequate trial for TRLLD; two or three different new antidepressants should be tried first.	42%

Category 1: Global definition and clinical presentation*Operational versus categorical definitions of TRLLD*	
If TRLLD is defined operationally, Stage I should be ‘failure of at least one adequate trial of a major antidepressant class.’ †	67%

Category 1: Global definition and clinical presentation*Number of treatment failures & psychotherapy*	
TRLLD should be defined as occurring in patients who do not achieve response, defined as a 50% or greater reduction in symptom severity from baseline. †	67%
In LLD, residual symptoms after two or three adequate treatment trials do not indicate TRLLD. †	67%

Category 1: Global definition and clinical presentation*Assessment tools*	
The Patient Health Questionnaire–9 (PHQ–9) is appropriate for monitoring symptoms in TRLLD.	58%
The Geriatric Depression Scale (GDS) is appropriate for monitoring symptoms in TRLLD.	62%

Category 2: Cognitive impairment, dementia, vascular depression	
Older adults with TRD, no prior depressive history, and comorbid MCI should be classified separately (e.g., Organic syndrome with TRD symptoms), not included in TRLLD.	42%
Older adults who meet criteria for vascular depression should be classified separately, not included in TRLLD.	37%

Category 3: Physical or mental comorbidities and TRLLD	
Chronic physical illnesses should be considered in the definition of TRLLD. †	67%

Category 4: Pharmacokinetics, pharmacodynamics, drug interactions and TRLLD	
When relevant, age-related pharmacokinetics and pharmacodynamics considerations should apply starting at age 60.	62%
TRLLD should not be diagnosed without checking plasma levels when a co-medication induces hepatic enzymes affecting antidepressant metabolism. †	67%
Medications with known depressogenic effects (e.g., beta-blockers) should be reduced or discontinued before labelling a case as TRLLD.	58%

Category 5: Treatment adherence, tolerance and TRLLD	
Adherence should be routinely assessed via plasma drug levels before diagnosing TRLLD.	58%

Category 6: Social, psychological factors and TRLLD	
Accessibility issues (e.g., access to ECT or blood level testing) should be considered in the definition of TRLLD. †	67%

*Note:* Statements listed here did not reach the predefined consensus threshold (≥70% agreement) among experts participating in the 2^nd^ SR (*n* = 24). Percentages represent the proportion endorsing each statement. Items marked with † did not reach consensus in the observed 2nd SR results but would cross the 70% threshold under the best-case scenario in the sensitivity analysis. See Supplementary Table S3.

**Table 6.** Distribution of responses to selected multiple-choice questions on key operational parameters relevant to the definition of TRLLD (2nd SR) Table 6. long description.

Question	Responses (*n*)	Responses (%)
What duration do you consider most appropriate to evaluate the response to antidepressants in late life? (optimal early decision point)		
4 weeks	11	46
6 weeks	10	42
≥8 weeks	3	13
From which week (e.g., 2 weeks…) can the effectiveness of antidepressant treatment be evaluated in a patient with LLD?		
< 4 weeks	5	21
4–6 weeks	14	58
> 6 weeks	5	21
From what age should late-onset TRLLD be defined?		
<60 years	2	8
60 years	7	29
≥65 years	12	50
≥70 years	2	8
Which scale or score do you *mostly use* in clinical practice for TRLLD?		
MADRS	11	46
GDS	6	25
HAM-D/HDRS	4	17
DSM criteria	1	4
HADS	1	4
Others (e.g., “clinical only”)	4	17
Please specify any tools (PHQ–9, GDS, HAM-D, Cornell) that should not be used for TRLLD.		
GDS	9	38
PHQ–9	8	33
Cornell	3	13
HAM-D	3	13
Others	2	8
When assessing TRLLD, which of the following should be prioritised?		
Symptom severity	11	46
Depending on the clinical context	11	46
Functional impairment	9	38

*Note:* This table summarises expert responses to predefined multiple-choice questions included in the 2nd SR (*n* = 24). For each question, the number (*n*) and percentage (%) indicate how many experts selected each response option. Some questions received multiple answers from experts.

**Table 7.** Summary of the free-text comments by thematic category in the 2nd SR Table 7. long description.

Category	Summary of comments from panel members
Category 1: Global definition and clinical presentation	One expert cautioned that the term treatment-resistant may be counterproductive if applied too early in the care pathway, as it may convey a defeatist message to patients, given that remission after a first antidepressant trial is uncommon.
Category 2: Co-occurrence of cognitive impairment, dementia, and vascular depression	Eight panellists emphasised the importance of distinguishing cognitive symptoms attributable to depression from mild cognitive impairment or dementia due to neurodegenerative aetiology.
Category 3: Co-occurrence of other physical and mental comorbidities	Some experts noted that physical conditions are highly prevalent in TRLLD and emphasised that physical and mental disorders may co-occur without requiring a single unifying explanatory framework. For definitional purposes, they argued that only aetiological factors supported by robust evidence as potential contributors to depressive symptoms should be retained.
Category 4: Pharmacokinetics, pharmacodynamics, and drug interactions	Comments diverged: some experts emphasised that clinical context should guide testing and interpretation, whereas others recommended that, in addition to plasma concentration monitoring, pharmacogenetic profiling should be considered.
Category 5: Treatment adherence and tolerance	Some experts noted that antidepressant plasma concentrations do not necessarily index adherence, as atypical levels may reflect pharmacogenetic variation. Several suggested that a detailed clinical history, supplemented by caregiver/family input, may be more informative than laboratory testing for sustained adherence, and called for greater attention to adverse effects.
Category 6: Social and psychological factors	The panel largely agreed that social isolation contributes to poorer treatment outcomes in LLD.

Overall, there was a high level of consensus on the contextual determinants relevant to the definition of TRLLD, while some items remained under debate. A best-case/worst-case sensitivity analysis for all 58 closed items was performed to assess the impact of the six non-responding experts in the 2nd SR. Results are presented separately for items that reached consensus in the observed analysis (Supplementary Table S2) and items that did not reach consensus (Supplementary Table S3). These tables show which consensus items would fall below the 70% threshold in the worst-case scenario, and which non-consensus items would reach the 70% threshold in the best-case scenario.

### Definition of TRLLD

**Figure 2.**
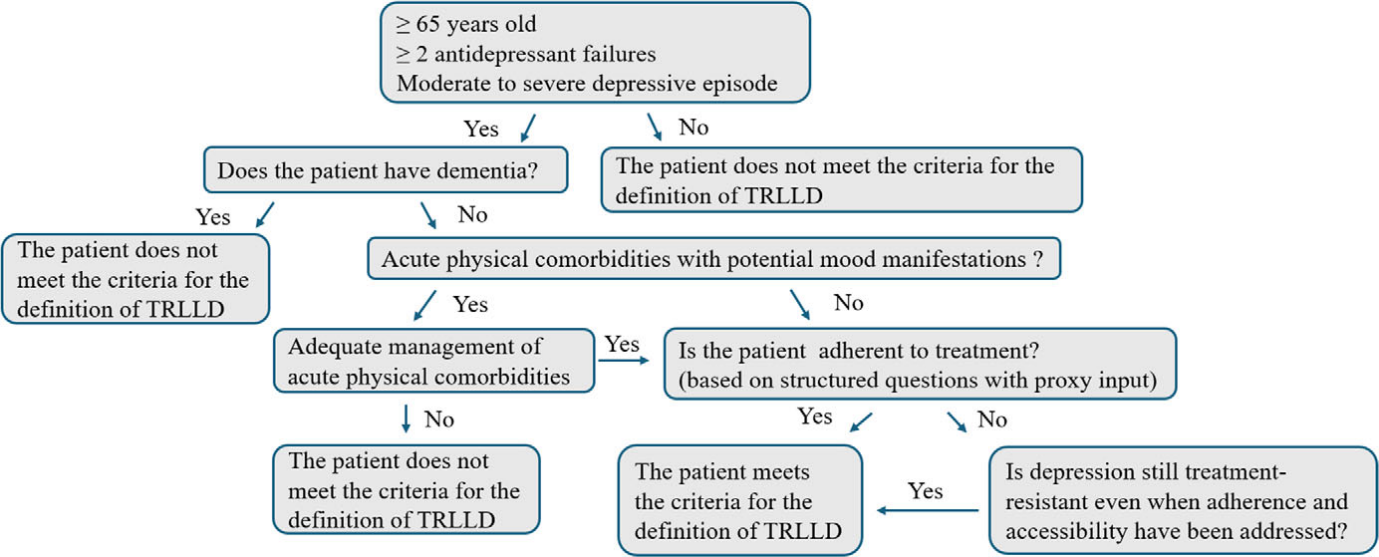
Consensus-based decision algorithm for defining TRLLD (categorical definition). *Note*: This algorithm operationalises the categorical TRLLD definition (minimum threshold), which corresponds to Stage II in the operational staging model presented in Table 9. It was derived from statements reaching consensus and was subsequently reviewed and refined by the expert panel in the 3rd SR. Figure 2. long description.

Based on information collected in the 1st and 2nd SR, we identified a set of contextual determinants relevant to the definition of TRLLD, which, while not incorporated as defining criteria, are highly relevant to implementation in routine practice, research design, and future evaluation of TRLLD. After synthesising the findings within each thematic category, the 3rd SR focused on consolidating the core definitional criteria of TRLLD into a practical categorical definition, an operational staging model, and a decision algorithm, presented in Tables 8 and 9; Figure 2.

**Table 8.** Summary of the expert panel’s consensus on the core definitional criteria for TRLLD (categorical definition) Table 8. long description.

Domain	Criterion	Clarifications/Notes
Global definition and symptoms	≥65 years old. ≥2 adequate antidepressant trial failures at guideline-supported doses and durations [34, 35]. A moderate to severe depressive episode as measured by a validated scale (e.g., MADRS ≥20 or HAM-D ≥17).	No upper age limit. Psychotherapy non-response is not part of the core TRLLD criterion, but it may count as one of three failed treatments in an operational definition and should be documented. The “optimal early decision point” for evaluating antidepressant response is after 4 weeks; this does not define the minimum duration of an adequate trial. Early-onset vs late-onset TRLLD should be indicated. First vs recurrent depressive episode should be indicated.
Cognitive comorbidities	Exclude patients with dementia from TRLLD classification.	MCI can be included in the TRLLD definition; cognitive follow-up should be ensured.
Physical comorbidities	Acute physical comorbidities with potential mood manifestations should be excluded.	Chronic physical comorbidities should be adequately managed.
Pharmacokinetics, pharmacodynamics, and treatment adherence	Structured questions about adherence with input from a proxy, such as a partner or caregiver.	
Social and Psychological factors	Access barriers (e.g., limited availability or treatment costs) should be documented and considered in clinical management, as they may limit the ability to complete adequate treatment trials.	

*Note*: This table summarises the core definitional criteria of the categorical TRLLD definition (minimum threshold), corresponding to Stage II in the operational staging model presented in Table 9. The statements presented in this table were synthesised from items reaching consensus and were subsequently reviewed and refined by the expert panel in the 3rd SR.

**Table 9.** Adaptation of the Thase and Rush staging model for TRLLD (European Task Force consensus) Table 9. long description.

Stage	Thase and Rush [18]	European Task Force TRLLD definition, 2026
Stage I	Failure of ≥1 adequate trial of antidepressant monotherapy.	Not endorsed as TRLLD (Stage I “failure of ≥1 adequate trial of a major antidepressant class,” did not reach consensus). Psychotherapy non-response does not count towards the ≥2 antidepressant trials, but may be documented as 1 of 3 failed treatments in an operational framework.
Stage II	Stage I + failure of adequate trial of a different antidepressant class.	Stage II (minimum TRLLD level): failure of ≥2 adequate antidepressant trials.
Stage III	Stage II + failure of adequate trial of a tricyclic antidepressant.	Stage III: Stage II + failure of augmentation therapy (e.g., aripiprazole, lithium).
Stage IV	Stage III + failure of adequate trial of a MAOI.	Stage IV: Stage III + failure of advanced intervention strategy (e.g., ECT, rTMS, ketamine/esketamine).
Stage V	Stage IV + failure of an ECT course.	Not applicable.

*Note:* In the European Task Force operational model, Stage II corresponds to the minimum categorical TRLLD definition and requires that all core definitional criteria specified in Table 8 are met. Stage I was not endorsed because failure of a single antidepressant trial did not meet the panel’s threshold for defining TRLLD. Stage II therefore represents the minimum categorical TRLLD definition, requiring failure of ≥2 adequate antidepressant trials. Stages III and IV were adapted to reflect contemporary LLD practice, informed by the Delphi panel responses to questions developed on the basis of our previous systematic review of interventions for TRLLD [14]: augmentation strategies are included at Stage III, and advanced interventions, including ECT, rTMS, and ketamine/esketamine, are included at Stage IV. The adapted staging model should be interpreted as a framework for describing the degree of treatment resistance in TRLLD rather than as a prescriptive treatment algorithm.

### Third survey round

In the 3rd SR, all expert panel members (who are co-authors of this consensus paper) reviewed, refined, and endorsed the final consensus and core definitional criteria in terms of the categorical definition, the operational staging model, and decision algorithm derived from the preceding SR (Tables 8 and 9; Figure 2).

## Discussion

This article reports a novel expert consensus on TRLLD, distinguishing core definitional criteria from consensus-endorsed contextual determinants identified across the three survey rounds. Based on the core criteria, we propose a final categorical definition, an operational staging model, and a decision algorithm for TRLLD (Tables 8 and 9; Figure 2).

Our categorical definition and operational staging model are built on the framework proposed by Patrick et al. [9]. They incorporate perspectives of a geographically diverse European expert panel, provide greater specificity and clarify how the severity and type of medical and cognitive comorbidities should influence classification, and propose an operational staging approach to TRLLD (Table 9).

### Global definition and clinical presentation

Consensus was reached on an age cutoff of ≥65 years for defining TRLLD. The age of 65 years is a conventional geriatric threshold and is also consistent with literature describing clinically relevant differences in depression in older adults. Meta-analytic evidence suggests that antidepressant response may be less robust in adults aged ≥65 years than in younger adult populations, supporting the need for age-specific consideration of treatment resistance from this age onward [26]. In addition, LLD is frequently characterised by greater medical comorbidity, vascular burden, neuroendocrine dysregulation, cognitive impairment, and increased risk of subsequent dementia, all of which may influence both clinical presentation and treatment response [27]. Therefore, the ≥65-year cutoff should be understood as a consensus-based and clinically pragmatic threshold [27]. No consensus was achieved on establishing a distinct “very-late-onset” subgroup beginning at age 85. There is an age-related increase in the incidence of new-onset depression across older age strata; however, incidence estimates specifically for individuals aged ≥85 years are less consistently reported [28, 29]. The depressive phenotype and response to treatment in this oldest group may differ from that observed in the broader older-adult population [30, 31]. Older individuals often present with somatic complaints, irritability, neurovegetative changes, and cognitive symptoms, rather than overt dysphoria [32]. This aligns with reviews describing ‘masked’ or atypical features, which can hinder the timely identification of TRLLD [32]. Further studies should clarify whether incident cases in the oldest-old have distinct features, particularly regarding treatment response, that might justify considering them as a separate category.

Within the panel, the MADRS was the most used monitoring instrument in clinical practice, followed by the GDS or HAM-D/HDRS. This pattern highlights a discrepancy: although atypical presentation was considered important, commonly used symptom scales do not fully capture atypical or masked manifestations. Consequently, these features were not included as core definitional criteria. This may lead to under-detection in routine clinical practice and requires further evaluation in future studies.

Most panellists agreed that non-response to two adequate antidepressant trials should be required, consistent with definitions applied in general adult populations [33]. As summarised in Table 8, although this Delphi study did not define specific target antidepressant doses or trial durations, adequacy should be judged according to recognised national and international guidelines, taking into account both dose and duration [34, 35]. In line with the principle of “start low, go slow, but go” [36], antidepressant treatment in older adults should be initiated and titrated cautiously, while aiming, when clinically appropriate and tolerated, for the same minimum effective doses as in younger adults unless contraindicated [37].

Consensus supported including older adults who develop late-life resistance to an antidepressant that was previously effective. Longitudinal data indicate heterogeneous trajectories in relation to treatment resistance: some patients show inadequate response from the first trial with an antidepressant, whereas others respond initially and then lose response over time [38]. These have been termed primary versus secondary treatment resistance, sometimes misinterpreted as early-onset and late-onset [39]. Distinguishing these trajectories is clinically salient in late life: secondary resistance may be more common in those with greater medical comorbidity and may be modifiable [40]. Additionally, there was strong consensus on distinguishing recurrent versus non-recurrent depression and early- versus late-onset forms – defined by the majority of the expert panel as depression that first manifests at age 65 years or older – given their potentially distinct aetiologies – and, consequently, different treatment implications [41, 42].

No consensus was reached on whether the mere presence of residual symptoms after two or three adequate treatment trials should independently qualify an individual as having TRLLD. Nonetheless, it is important to emphasise that minimising residual symptoms remains a central therapeutic goal. In later life, these symptoms are common and should be actively evaluated and managed, while also recognising that some may reflect underlying physical illness, frailty, or other non-depressive processes requiring targeted assessment and treatment [43]. Where residual symptoms are attributable to ongoing depressive pathology, they are strongly associated with increased relapse risk and poorer quality of life [44, 45].

Experts endorsed the need for a categorical approach to TRLLD and agreed that the Thase and Rush (1997) [18] staging model should be adapted beginning at Stage II. Stage I – defined as failure of a single adequate antidepressant trial – did not reach consensus, whereas Stage II corresponds to the minimum threshold of the categorical TRLLD definition established in this study (Table 8).

The adapted staging model should be interpreted as a framework for describing the degree of treatment resistance in TRLLD rather than as a prescriptive treatment algorithm. The design of the survey questions addressing this model was informed by the findings of our preceding systematic review [14]. The differences from the original Thase and Rush model reflect the responses of the Delphi panel across the 2nd SR (see Table 4).

One expert cautioned that the label treatment-resistant can convey a defeatist approach if applied too early in care [46], which is especially important given the high non-response rates in LLD, approximately 51% after one antidepressant trial [6]. This concern is further contextualised by meta-analytic evidence concluding that, in adults aged ≥65 years, the overall benefit of second-generation antidepressants (e.g., SSRIs, SNRIs, or mirtazapine) is modest and heterogeneous, and potentially attenuated in the context of frailty and tolerability issues [26, 47]. To mitigate this risk, it is important to reiterate that the TRLLD designation only applies after two adequate antidepressant treatments have been unsuccessful, and that there are still multiple possible and potentially effective therapeutic avenues available [14, 35] when following an evidence-based, guideline-informed sequential treatment approach.

In terms of psychotherapy, 71% of experts indicated that non-response to psychotherapy should not count as a failed treatment in the categorical definition of TRLLD. Although this met the predefined consensus threshold, it was one of the weakest consensus findings and did not remain robust in the sensitivity analyses. However, these analyses should be interpreted cautiously, as most panellists with formal psychotherapy training were retained for the 2nd SR. In the proposed definition, psychotherapy non-response was not retained as a core definitional criterion, but its role should remain open to further evaluation and may be documented within the broader operational staging framework. The lack of consensus also likely reflects the limited and heterogeneous evidence base specific to TRLLD, which complicates operationalisation (e.g., modality, intensity, delivery format). It may also be influenced by variability in access to and delivery of psychotherapeutic services for older adults across health systems. Notably, scientific evidence of LLD emphasises the importance of combining pharmacotherapy with evidence-based psychotherapies wherever possible, while acknowledging that research into psychotherapy for TRLLD remains limited [14, 48].

Experts agreed that psychotherapy may be included as one of three failed treatments in an operational definition. Therefore, although treatment resistance is established by the failure of two adequate antidepressant treatments, a history of non-response to psychotherapy should be mentioned explicitly (Table 9).

Most panellists agreed that one should wait approximately 4 weeks to observe the effects of antidepressants. This stands in contrast to literature suggesting that, in patients aged ≥65 years, a longer trial than in general adult populations may be warranted (8 weeks) [13]. As endorsed by most panel members, waiting 8 weeks is often not feasible in real-world clinical practice, underscoring the need for close monitoring and timely, proactive adjustments when early improvement is absent. In this context, the panel’s preference for week 4 as an optimal early decision point is consistent with the literature on early decision-making in LLD, which suggests that (i) more than 40% of partial responders at week 4 ultimately attain full response by the end of acute treatment; (ii) fewer than 25% of non-responders at week 4 attain full response by week 12; and (iii) early full response is largely sustained through week 12 [49–51]. Accordingly, changing treatment before week 4 may lead to premature discontinuation of potentially effective therapy in too many patients, whereas the absence of at least partial response by week 4 may reasonably prompt treatment modification (e.g., switch or augmentation).

### Cognitive impairment and TRLLD

The panel agreed that individuals with dementia should be excluded from the definition of TRLLD and emphasised the importance of determining the aetiology of cognitive decline in older adults with TRLLD. Even if biomarkers are not part of the TRLLD definition, some could be part of the differential diagnosis. Emerging blood-based biomarkers such as plasma p-tau217 or neurofilament light (NfL), markers of specific Alzheimer pathology and non-specific neurodegeneration, respectively, may provide adjunctive evidence to distinguish whether cognitive symptoms in TRLLD reflect a primary neurodegenerative process or are secondary to depression [52–55]. However, particularly in the very old, abnormal tau measures may also reflect preclinical pathology and may not map directly onto current symptoms, so results should be interpreted in a clinical context [56, 57]. Where available and clinically indicated, additional investigations such as amyloid PET imaging for Alzheimer’s disease or cerebrospinal fluid α-synuclein assays for Lewy body disease may further support differential diagnosis and phenotyping [58]. The lack of consensus on defining patients with TRLLD, no prior depressive history, and comorbid mild cognitive impairment (MCI) as a separate category may reflect the aetiological heterogeneity of MCI in LLD. While cognitive impairment may be at least partly related to the depressive episode in some patients [59], in others, it may indicate an underlying neurodegenerative process [60, 61]. Therefore, patients with TRLLD and comorbid cognitive impairment should receive longitudinal cognitive follow-up [61]. From a clinical standpoint, individuals with LLD/TRLLD and cognitive impairment, irrespective of the underlying aetiology, require active treatment, as they may be particularly susceptible to accelerated brain ageing and adverse outcomes [10, 62].

Apathy is another frequent and clinically meaningful feature in LLD. In a geriatric cohort, approximately 38% of older adults with depression have clinically significant apathy, and its severity predicts disability independently of overall depressive severity [63]. Importantly, apathy is not a formal diagnostic criterion for a depressive episode in DSM-5 or ICD-10; therefore, its presence or absence should not be used to determine whether the minimum severity threshold (i.e., a moderate depressive episode) is met. Nonetheless, apathy remains clinically relevant in LLD and should be assessed and documented as an associated feature.

### Other comorbidities and TRLLD

Some experts questioned whether residual somatic symptoms (e.g., sleep, appetite, fatigue/energy-related symptoms) should count against the TRLLD definition. Ultimately, the statement that “residual symptoms after two or three adequate treatments indicate TRLLD” did not reach consensus, so residual somatic symptoms were not included as a defining criterion. Nonetheless, the clinical management of somatic residual symptoms remains understudied, and further research should define optimal strategies for their assessment and treatment.

Consensus was reached on the need to screen for physical pathology before labelling a case as TRLLD. Importantly, in individuals with high somatic burden, comorbidity is often associated with lower treatment adherence [64]. Accordingly, these patients warrant closer adherence monitoring to minimise the risk of misclassifying non-response due to poor adherence as TRLLD. Furthermore, there was consensus to exclude from the definition patients with depressive symptoms occurring in the context of another primary psychiatric disorder (e.g., bipolar disorder or schizoaffective disorder). Further discussions by task force groups are needed to determine whether certain resistance criteria defined for bipolar depression in adults can be adapted to the late-life population [65].

### Treatment adherence and tolerance and TRLLD

Our panel agreed that TRLLD assessment should include structured questions about adherence, with input from a proxy such as a partner or carer. This approach can be difficult to implement for socially isolated patients and may also be perceived as intrusive; therefore, it should only be undertaken with the patient’s consent. Nevertheless, it remains clinically relevant, as adherence in older adults with depression is often suboptimal.

Studies in primary care cohorts of older adults show that approximately 13.5% do not commence prescribed antidepressants, 15.2% adhere to treatment insufficiently, and 37.1% do not persist with treatment during roughly the first year [66]. In geriatric cohorts, about one-half of older adults achieve an adherence level of at least 80% of days covered [67]. These findings highlight the clinical importance of structured adherence assessment and, where appropriate, corroborating adherence with proxy input (e.g., a partner or caregiver).

The use of plasma antidepressant levels to monitor adherence was one of the most contentious issues. Some panel members highlighted the limited availability of such testing in most centres and the lack of robust evidence for a direct correspondence between plasma concentrations and adherence, whereas others argued that this relatively inexpensive assay could become more accessible if incorporated into routine practice [68].

The British Association for Psychopharmacology’s guidelines for the treatment of depressive disorders emphasise the need for effective strategies to enhance medication adherence [69]. They also note that therapeutic drug monitoring can help assess treatment adherence and lack of efficacy at apparently adequate doses [69].

The authors emphasise that aligning treatment with patient preferences and implementing structured follow-up plans improves both adherence and outcome; that adherence counselling is beneficial, whereas information leaflets alone are not; and that once-daily regimens can be as effective as multiple daily dosing and are associated with better adherence [69]. Psychoeducational interventions can improve outcomes in later life [70].

Further studies should determine the proportion of patients classified as resistant under the presented definition who exhibit non-adherence or subtherapeutic plasma antidepressant levels.

### Social and psychological factors and TRLLD

Finally, the panel reached consensus on the relevance of social determinants in both the definition and the care pathway for TRLLD. Experts agreed that social isolation contributes to poorer treatment outcomes in LLD and that access barriers should be documented and considered when interpreting treatment adequacy and resistance. Costs and insurance coverage limitations restrict pharmacological access in LLD, affecting prescription initiation, continuity, and adherence [71, 72]. These factors are contextual determinants relevant to research and clinical implementation, but should not be considered core criteria for defining TRLLD. Addressing these barriers, through facilitated access to medications, financial and logistical support, and collaborative-care models embedded in primary care, is essential. Furthermore, social isolation can restrict access to evidence-based treatments –including pharmacotherapy, psychotherapy, or tele–mental health– and may shape how TRLLD is identified in routine clinical practice. Despite recent improvements in digital connectivity, substantial access gaps persist among socially disadvantaged, rural, and homebound older adults, limiting engagement in remote interventions and exacerbating inequities [73, 74].

### Sensitivity analysis of consensus findings

A best-case/worst-case sensitivity analysis was performed to assess the potential impact of the six experts who did not complete the 2nd SR. This analysis showed that several items close to the 70% consensus threshold were sensitive to assumptions regarding non-responders. These findings do not invalidate the 2nd SR results, which were based on the prespecified Delphi sample of 24 respondents, but they should be considered when interpreting the robustness of individual items. Full item-level results are provided in Supplementary Tables S2 and S3.

### Strengths and limitations

This study applies a rigorous Delphi methodology aligned with CREDES guidance (Supplementary Table S1) [25], using iterative SR, prespecified consensus thresholds, and structured feedback between SR. The panel was international and multidisciplinary, providing broad clinical input; however, geographical representation was uneven, with a substantial proportion of participants from France, England, and Switzerland. This geographical imbalance may limit the representativeness of the panel and should therefore be considered when interpreting the findings. Item generation was anchored in a comprehensive literature review and refined through open-ended responses (1st SR), enhancing content validity. The process achieved substantial agreement (consensus on 72.4% of closed items) and produced operational outputs, a consensus definition, and a decision algorithm that are readily applicable to practice.

In terms of limitations, the panel included only one psychologist and no psychosocial practitioners, which may have reduced the robustness of consensus on psychotherapy-related items and calls for cautious interpretation in the absence of broader specialist input. This should, however, be considered alongside the fact that the 2nd SR included five psychiatrists from Germany and Switzerland, where the official specialist designation explicitly combines psychiatry and psychotherapy [75, 76]. Nevertheless, broader multidisciplinary input, particularly from clinical psychologists and psychotherapy specialists, should be considered in future revisions of the TRLLD definition. More generally, and inherent to the Delphi approach, these findings reflect expert consensus rather than empirical testing; consensus represents agreement among panellists and does not necessarily indicate an objectively “correct” position, while persistent disagreement remains informative. Therefore, future studies should evaluate the proposed TRLLD criteria in independent samples by examining their feasibility in routine practice, inter-rater reliability, and clinical validity. The high proportion of closed questions in the 2nd SR may have constrained expression of nuance, and anchoring effects may have arisen because panellists viewed summaries from previous SR. To mitigate this issue, a free-text field for open comments was included in each category. Another limitation is that not all non-pharmacological approaches were considered in shaping the definition. Some, such as psychotherapy, were considered in the Delphi process. However, other interventions – like physical exercise – with a growing body of evidence supporting their role in TRLLD [77–79], were not specifically included.

Finally, while the panel achieved consensus on the importance of adherence monitoring and age-specific pharmacokinetic considerations, practical implementation strategies – including the role and timing of therapeutic drug monitoring, approaches to managing complex drug–drug interactions, and systematic assessment of sickness behaviour in the context of medical comorbidity – were not fully operationalised and require further empirical investigation.

## Conclusion

We present an expert-derived consensus definition for TRLLD, developed to address the unique challenges in older adults. The proposed criteria offer a practical framework to enhance diagnostic vigilance, promote timely intervention, and harmonise clinical and research practices. These criteria are deliberately conservative to avoid premature “treatment-resistant” labelling. By standardising definitions and pathways, this framework may also facilitate epidemiological understanding and improve the identification and enrolment of patients in research studies. Future priorities include the development of prevention strategies and targeted interventions informed by clinical trials, as well as prospectively validating this definition across diverse care settings. Another priority is testing the prognostic validity of this definition against clinical outcomes and treatment trajectories.

## Supporting information

10.1192/j.eurpsy.2026.12228.sm001 Pozuelo Moyano et al. supplementary material Pozuelo Moyano et al. supplementary material

## Data Availability

All data generated or analysed during this study are included in this published article.

## Supplementary material

The supplementary material for this article can be found at http://doi.org/10.1192/j.eurpsy.2026.12228.

## Long descriptions

### [Table 1] Long description

The table consists of two columns: Selection criteria and How it was implemented in this study.

Row 1: Identifying the most appropriate categories of experts for the panel. Implementation: Identified experts in old-age psychiatry, geriatrics, psychology, neurology, and related fields with expertise in L L D. Experts were selected for clinical or research experience. Recruitment aimed for broad European geographical representation to enrich perspectives on T R L L D.

Row 2: Identifying experts. Implementation: Names compiled from previous research, publications on L L D or T R L L D, and membership in professional associations. Most panellists were independent academic clinicians with no conflicts of interest. Coordination was by a steering committee of independent researchers.

Row 3: Contacting individuals. Implementation: Experts were invited to nominate additional professionals via a snowball sampling approach to ensure a diverse pool of expertise.

Row 4: Creating sub-lists for each stakeholder group. Implementation: Experts were grouped by professional background, including psychology, geriatrics, psychiatry, neurology, and old-age psychiatry.

Row 5: Inviting the experts. Implementation: The study aimed to assemble a convenience sample of at least 30 experts.

Navigate back to [Table 1].

### [Figure 1] Long description

The flowchart is structured with a central column of process boxes, a vertical timeline arrow on the right, and summary labels on the left.

Timeline and Phases:

- 2024: Literature review and initial framework development.

- January - February 2025: Steering Committee established and selection of experts. 30 invited and 30 accepted.

- March - April 2025: 1st Survey Round. This phase involves 6 open-ended questions on contextual determinants of T R L L D. Steps include pre-testing by the Steering Committee, volunteer psychiatrists, and research assistants; collection of responses from 30 out of 30 experts; and feedback sent via email.

- May - July 2025: 2nd Survey Round. This phase involves 70 closed-ended yes or no questions on contextual determinants of T R L L D. Steps include pre-testing by the same group; collection of responses from 24 out of 30 experts; and feedback sent via email.

- August - December 2025: 3rd Survey Round. This phase focuses on core definitional criteria for T R L L D. Steps include categorical definition, operational staging model, and derived decision algorithm; Steering Committee review; and expert panel review with 30 out of 30 experts participating.

- January 2026: Final validation.

The vertical arrow on the right spans the entire duration, pointing downward to indicate the progression of time.

Navigate back to [Figure 1].

### [Table 2] Long description

The table consists of four columns: Item, 1st S R n equals 30, 2nd S R n equals 24, and Experts not completing 2nd S R n equals 6.

Primary professional background:

- Psychiatry general: 16 53.3 percent, 13 54.2 percent, 3 50.0 percent.

- Old age psychiatry: 10 33.3 percent, 7 29.2 percent, 3 50.0 percent.

- Geriatrics: 2 6.7 percent, 2 8.3 percent, 0 0.0 percent.

- Neurology: 1 3.3 percent, 1 4.2 percent, 0 0.0 percent.

- Psychology: 1 3.3 percent, 1 4.2 percent, 0 0.0 percent.

Years of professional experience:

- 6 to 9 years: 3 10.0 percent, 3 12.5 percent, 0 0.0 percent.

- 10 to 20 years: 12 40.0 percent, 12 50.0 percent, 0 0.0 percent.

- Greater than 20 years: 15 50.0 percent, 9 37.5 percent, 6 100.0 percent.

Clinical degree or academic background:

- M D no P h D: 11 36.7 percent, 9 37.5 percent, 2 33.3 percent.

- P h D with or without M D: 19 63.3 percent, 15 62.5 percent, 4 66.7 percent.

Country of participants:

- Belgium: 1 3.3 percent, 1 4.2 percent, 0 0.0 percent.

- England: 6 20.0 percent, 4 16.7 percent, 2 33.3 percent.

- France: 7 23.3 percent, 5 20.8 percent, 2 33.3 percent.

- Germany: 1 3.3 percent, 1 4.2 percent, 0 0.0 percent.

- Italy: 1 3.3 percent, 1 4.2 percent, 0 0.0 percent.

- Monaco: 1 3.3 percent, 0 0.0 percent, 1 16.7 percent.

- Netherlands: 1 3.3 percent, 1 4.2 percent, 0 0.0 percent.

- Norway: 1 3.3 percent, 1 4.2 percent, 0 0.0 percent.

- Poland: 1 3.3 percent, 1 4.2 percent, 0 0.0 percent.

- Portugal: 1 3.3 percent, 1 4.2 percent, 0 0.0 percent.

- Romania: 1 3.3 percent, 1 4.2 percent, 0 0.0 percent.

- Spain: 2 6.7 percent, 2 8.3 percent, 0 0.0 percent.

- Sweden: 1 3.3 percent, 1 4.2 percent, 0 0.0 percent.

- Switzerland: 5 16.7 percent, 4 16.7 percent, 1 16.7 percent.

Navigate back to [Table 2].

### [Table 3] Long description

The table consists of a single column titled Question. The rows contain the following six questions. One. How would you define treatment-resistant late-life depression TRLLD? Two. How many ineffective treatment trials should be required to define treatment resistance? Three. Which classes of treatments should be considered when evaluating an unsuccessful attempt to treat late-life depression? Four. Do you think unsuccessful psychological treatments should be included when defining TRLLD? Five. Should the biological causes and mechanisms of disease investigations be included when defining TRLLD? Six. Are there any criteria for defining treatment-resistant depression that should be specifically considered in the older population compared with the adult population?

Navigate back to [Table 3].

### [Table 4] Long description

The table is divided into six categories.

Category 1: Global definition and clinical presentation.

- Age definition and diagnostic criteria: Age-specific TRLLD criteria 92 percent. Defining TRLLD as 65 years and older 79 percent. Inclusion of age 85 or older 83 percent. Minimum severity threshold 75 percent. Threshold as moderate depressive episode 90 percent. Specifying early-onset versus late-onset 96 percent. Distinguishing recurrent versus first episode 83 percent. Including resistance to previously effective antidepressants 88 percent.

- Operational versus categorical definitions: Defined operationally using a continuum 83 percent. Adapting existing TRD staging models 83 percent. Stage 2 failure of two trials from distinct classes 78 percent. Stage 3 failure of augmentation 83 percent. Stage 4 failure of EC T, r TM S, or ketamine 71 percent. Operational definition for research 88 percent. Model should not begin with psychotherapy at Stage 1 75 percent.

- Number of treatment failures and psychotherapy: Failure to respond to two trials 83 percent. Psychotherapy not counted as one failed treatment 71 percent. Psychotherapy defined as one out of three failed treatments 82 percent.

- Treatment duration: Specific minimum duration necessary 100 percent. 10 to 12 weeks considered too long for clinical practice 88 percent. Start low, go slow, but go principle 92 percent.

- Symptom presentation: Include atypical and masked symptoms 92 percent. Consider differences between clinician and patient reports 96 percent. Consider discrepancies between symptom reduction and functional improvement 96 percent.

- Assessment tools: MADRS 88 percent. HAM dash D 88 percent. Cornell Scale for Depression in Dementia 79 percent.

Category 2: Cognitive impairment, dementia, vascular depression.

- Cognitive impairment interferes with diagnosis 96 percent. Affects treatment response 100 percent. Routine cognitive screening 92 percent. Excluding those with dementia and no prior history 75 percent. Apathy as only remaining symptom does not indicate TRLLD 75 percent.

Category 3: Physical or mental comorbidities.

- Screening for thyroid, testosterone, or vitamin deficiencies 92 percent. Primary psychiatric disorders do not indicate TRLLD 83 percent. Managing physical comorbidities like chronic pain 79 percent.

Category 4: Pharmacokinetics, pharmacodynamics, drug interactions.

- Age-specific characteristics should be considered 96 percent.

Category 5: Treatment adherence and tolerance.

- Poor adherence as common cause of failure 96 percent. Discontinuation due to side effects is not a failed trial 92 percent. Structured questions about adherence with proxy input 83 percent. Additional monitoring for those with cognitive impairment 88 percent.

Category 6: Social and psychological factors.

- Social isolation contributes to poor outcomes 92 percent. Routine assessment of psychosocial stressors 83 percent.

Navigate back to [Table 4].

### [Table 5] Long description

The table is organized into six categories with associated statements and their expert agreement percentages (ranging from 37% to 67%).

* Category 1: Global definition and clinical presentation. Includes age definition and diagnostic criteria (62% agreement on adult TRD criteria applicability; 58% on age 85 as a distinct subgroup; 58% on antidepressant trial requirements in recurrent depression). Operational versus categorical definitions (67% on Stage I definition). Number of treatment failures and psychotherapy (67% on 50% symptom reduction threshold; 67% on residual symptoms after 2 or 3 trials). Assessment tools (58% for PHQ 9; 62% for GDS).

* Category 2: Cognitive impairment, dementia, vascular depression. (42% on classifying MCI separately; 37% on classifying vascular depression separately).

* Category 3: Physical or mental comorbidities. (67% on considering chronic physical illness in the definition).

* Category 4: Pharmacokinetics, pharmacodynamics, drug interactions. (62% on age 60 threshold; 67% on checking plasma levels for hepatic enzyme induction; 58% on reducing depressogenic medications).

* Category 5: Treatment adherence and tolerance. (58% on routine plasma drug level assessment).

* Category 6: Social and psychological factors. (67% on considering accessibility issues like ECT access).

Navigate back to [Table 5].

### [Table 6] Long description

A table with three columns: Question, Responses n, and Responses percent. It details the following survey results from 24 experts:

* For the appropriate duration to evaluate antidepressant response: 4 weeks (11 responses, 46 percent), 6 weeks (10 responses, 42 percent), and greater than or equal to 8 weeks (3 responses, 13 percent).

* For the week when effectiveness can first be evaluated: less than 4 weeks (5 responses, 21 percent), 4 to 6 weeks (14 responses, 58 percent), and greater than 6 weeks (5 responses, 21 percent).

* For the age to define late-onset TRLLD: less than 60 years (2 responses, 8 percent), 60 years (7 responses, 29 percent), greater than or equal to 65 years (12 responses, 50 percent), and greater than or equal to 70 years (2 responses, 8 percent).

* For clinical scales mostly used: MADRS (11 responses, 46 percent), GDS (6 responses, 25 percent), HAM dash D or HDRS (4 responses, 17 percent), D S M criteria (1 response, 4 percent), H A D S (1 response, 4 percent), and others (4 responses, 17 percent).

* For tools that should not be used: GDS (9 responses, 38 percent), PHQ dash 9 (8 responses, 33 percent), Cornell (3 responses, 13 percent), HAM dash D (3 responses, 13 percent), and others (2 responses, 8 percent).

* For priorities when assessing TRLLD: Symptom severity (11 responses, 46 percent), Depending on the clinical context (11 responses, 46 percent), and Functional impairment (9 responses, 38 percent).

Navigate back to [Table 6].

### [Table 7] Long description

The table consists of two columns: Category and Summary of comments from panel members.

* Category 1: Global definition and clinical presentation. Summary: One expert cautioned that the term treatment-resistant may be counterproductive if applied too early, as it may convey a defeatist message since remission after a first antidepressant trial is uncommon.

* Category 2: Co-occurrence of cognitive impairment, dementia, and vascular depression. Summary: Eight panellists emphasized distinguishing cognitive symptoms of depression from mild cognitive impairment or dementia due to neurodegenerative etiology.

* Category 3: Co-occurrence of other physical and mental comorbidities. Summary: Experts noted physical conditions are prevalent in TRLLD and may co-occur without a single unifying framework. Only etiological factors with robust evidence should be retained for definitions.

* Category 4: Pharmacokinetics, pharmacodynamics, and drug interactions. Summary: Comments diverged between guiding testing by clinical context and recommending plasma concentration monitoring alongside pharmacogenetic profiling.

* Category 5: Treatment adherence and tolerance. Summary: Experts noted plasma concentrations may reflect pharmacogenetic variation rather than adherence. They suggested clinical history and caregiver input are more informative than laboratory testing and called for attention to adverse effects.

* Category 6: Social and psychological factors. Summary: The panel agreed that social isolation contributes to poorer treatment outcomes in LL D.

Navigate back to [Table 7].

### [Table 8] Long description

The table is organized into three columns: Domain, Criterion, and Clarifications/Notes.

* Domain: Global definition and symptoms.

- Criterion: Age greater than or equal to 65 years old; greater than or equal to 2 adequate antidepressant trial failures at guideline-supported doses and durations; a moderate to severe depressive episode measured by a validated scale such as MADRS greater than or equal to 20 or HAM dash D greater than or equal to 17.

- Clarifications/Notes: No upper age limit. Psychotherapy non-response is not a core criterion but may count as one of three failed treatments. The optimal early decision point for response is 4 weeks. Early-onset versus late-onset and first versus recurrent episodes should be indicated.

* Domain: Cognitive comorbidities.

- Criterion: Exclude patients with dementia from TRLLD classification.

- Clarifications/Notes: MCI can be included; cognitive follow-up should be ensured.

* Domain: Physical comorbidities.

- Criterion: Acute physical comorbidities with potential mood manifestations should be excluded.

- Clarifications/Notes: Chronic physical comorbidities should be adequately managed.

* Domain: Pharmacokinetics, pharmacodynamics, and treatment adherence.

- Criterion: Structured questions about adherence with input from a proxy, such as a partner or caregiver.

- Clarifications/Notes: None provided.

* Domain: Social and psychological factors.

- Criterion: Access barriers such as limited availability or treatment costs should be documented and considered in clinical management.

- Clarifications/Notes: None provided.

Navigate back to [Table 8].

### [Table 9] Long description

The table consists of three columns: Stage, Thase and Rush, and European Task Force TRLLD definition, 2026.

* Stage I: Thase and Rush define this as failure of 1 or more adequate trials of antidepressant monotherapy. The European Task Force does not endorse this as TRLLD. They note that psychotherapy non-response does not count toward the 2 required antidepressant trials but may be documented as 1 of 3 failed treatments.

* Stage II: Thase and Rush define this as Stage I plus failure of an adequate trial of a different antidepressant class. The European Task Force defines this as the minimum TRLLD level, requiring failure of 2 or more adequate antidepressant trials.

* Stage III: Thase and Rush define this as Stage II plus failure of an adequate trial of a tricyclic antidepressant. The European Task Force defines this as Stage II plus failure of augmentation therapy, such as aripiprazole or lithium.

* Stage IV: Thase and Rush define this as Stage III plus failure of an adequate trial of a MAOI. The European Task Force defines this as Stage III plus failure of an advanced intervention strategy, such as EC T, r T M S, or ketamine or esketamine.

* Stage V: Thase and Rush define this as Stage IV plus failure of an ECT course. The European Task Force marks this as Not applicable.

Navigate back to [Table 9].

### [Figure 2] Long description

The flowchart begins at the top with a primary entry box containing three criteria: greater than or equal to 65 years old, greater than or equal to 2 antidepressant failures, and moderate to severe depressive episode.

If these criteria are not met (No), the path leads to a box stating: The patient does not meet the criteria for the definition of TRLLD.

If met (Yes), the next question is: Does the patient have dementia?

- If Yes, the patient does not meet the criteria for the definition of TRLLD.

- If No, the next question is: Acute physical comorbidities with potential mood manifestations?

From the physical comorbidities question:

- If Yes, the path leads to: Adequate management of acute physical comorbidities. If this management is not achieved (No), the patient does not meet the criteria. If it is achieved (Yes), it joins the No path from the previous question.

- If No, or after successful management, the next question is: Is the patient adherent to treatment? (based on structured questions with proxy input).

From the adherence question:

- If Yes, the patient meets the criteria for the definition of TRLLD.

- If No, the final question is: Is depression still treatment-resistant even when adherence and accessibility have been addressed? If Yes, the patient meets the criteria for the definition of TRLLD.

Navigate back to [Figure 2].
