# Supplementary material for: Definition of treatment-resistant late-life depression: Conclusions from a European Task Force Delphi process
Source: Eur Psychiatry. 2026 Jun 2;69(1):e68. doi: 10.1192/j.eurpsy.2026.12228 (PMC13359010; doi:10.1192/j.eurpsy.2026.12228)
Supplement: Pozuelo Moyano et al. supplementary material [file S0924933826122287sup001.zip › Supplementary Table S2.docx]

**Supplementary Table S2. Sensitivity analysis of Round 2 agreement percentages under worst-case and best-case assumptions for non-responders.**

Observed percentages are based on experts participating in Round 2 (n = 24). Worst-case and best-case percentages assume that all six non-responding experts disagreed or agreed, respectively, and are recalculated over the full panel (n = 30). The last column shows the average of the worst-case and best-case values. Items marked with one asterisk (*) fell below the 70% consensus threshold under the worst-case scenario, assuming that all six experts who did not complete the 2nd SR would have disagreed with the item. Items marked with two asterisks (**) also fell below the 70% threshold when considering the midpoint between the worst-case and best-case estimates. In the best-case scenario, all six non-responding experts were assumed to agree with the item.

| Category 1: Global definition and clinical presentation  Age definition & diagnostic criteria | **Observed** | **Worst-case** | **Best-case** | **Average of the worst/best** |
| --- | --- | --- | --- | --- |
| Age-specific TRLLD criteria should be developed to better reflect the needs of older populations. | 92.00% | 73.60% | 93.60% | 83.60% |
| It is important to define TRLLD as applying to individuals aged 65 years and older. * | 79.00% | 63.20% | 83.20% | 73.20% |
| Individuals with the onset of treatment-resistant depression (TRD) at age 85 or older (≥85) should not be excluded from the definition of TRLLD. * | 83.00% | 66.40% | 86.40% | 76.40% |
| There should be a minimum severity threshold for diagnosing TRLLD. * | 75.00% | 60.00% | 80.00% | 70.00% |
| The minimum severity threshold for diagnosing TRLLD should be moderate depressive episode. | 90.00% | 72.00% | 92.00% | 82.00% |
| The definition of TRLLD should specify whether it refers to early-onset or late-onset TRLLD. | 96.00% | 76.80% | 96.80% | 86.80% |
| The distinction between recurrent depressive disorder and a first episode LLD should be specified in the definition of TRLLD. * | 83.00% | 66.40% | 86.40% | 76.40% |
| TRLLD should include cases where older adults with a history of recurrent depressive disorder develop resistance to an antidepressant that was previously effective. | 88.00% | 70.40% | 90.40% | 80.40% |
| Category 1: Global definition and clinical presentation  Operational versus categorical definitions of TRLLD | | | | |
| TRLLD should be defined operationally—rather than as a fixed category—by using a continuum or staging model based on the number and type of failed treatment attempts [18]. * | 83.00% | 66.40% | 86.40% | 76.40% |
| Existing TRD staging models [18] should be adapted for older adults. * | 83.00% | 66.40% | 86.40% | 76.40% |
| If TRLLD should be defined operationally, the following TRLLD model should be used: Stage II: Failure of two adequate trials from two distinct classes. * | 78.00% | 62.40% | 82.40% | 72.40% |
| If TRLLD should be defined operationally, the following TRLLD model should be used: Stage III: Stage II plus failure of augmentation (e.g., aripiprazole, lithium, methylphenidate). * | 83.00% | 66.40% | 86.40% | 76.40% |
| If TRLLD should be defined operationally, the following TRLLD model should be used: Stage IV: Stage III plus failure of Electroconvulsive Therapy (ECT), repetitive Transcranial Magnetic Stimulation (rTMS), or ketamine. ** | 71.00% | 56.80% | 76.80% | 66.80% |
| In research, TRLLD should also be defined operationally rather than as a fixed category. | 88.00% | 70.40% | 90.40% | 80.40% |
| If TRLLD should be defined operationally, the model should not begin with psychotherapy at Stage I. * | 75.00% | 60.00% | 80.00% | 70.00% |
| Category 1: Global definition and clinical presentation  Number of treatment failures & psychotherapy | | | | |
| TRLLD definition should include a failure to respond to two adequate antidepressant trials. * | 83.00% | 66.40% | 86.40% | 76.40% |
| Psychotherapeutic interventions should not be counted as one failed treatment when defining TRLLD. ** | 71.00% | 56.80% | 76.80% | 66.80% |
| Psychotherapy interventions should be defined as one out of three failed treatments used to define TRLLD. * | 82.00% | 65.60% | 85.60% | 75.60% |
| Category 1: Global definition and clinical presentation  Treatment duration and evaluation timeline | | | | |
| A specific minimum duration for an antidepressant trial in older adults is necessary to adequately assess treatment response in LLD. | 100.00% | 80.00% | 100.00% | 90.00% |
| Although the literature suggests that antidepressant response in older patients may require 10–12 weeks, this duration is considered too long for the demands of real-world clinical practice. | 88.00% | 70.40% | 90.40% | 80.40% |
| Clinicians should follow the principle “start low, go slow, but go,” using, if needed, the same minimum effective doses as in younger adults unless contraindicated. | 92.00% | 73.60% | 93.60% | 83.60% |
| Category 1: Global definition and clinical presentation  Symptom presentation in older adults | | | | |
| The diagnostic criteria for TRLLD should include atypical and masked symptoms commonly observed in older adults. | 92.00% | 73.60% | 93.60% | 83.60% |
| The differences between improvements observed by clinicians and reported by patients should be considered when defining TRLLD. | 96.00% | 76.80% | 96.80% | 86.80% |
| Discrepancies between symptom reduction and functional improvement should be considered in the TRLLD definition. | 96.00% | 76.80% | 96.80% | 86.80% |
| Category 1: Global Definition and Clinical Presentation  Assessment tools | | | | |
| The Montgomery–Åsberg Depression Rating Scale (MADRS) is an appropriate tool for monitoring symptoms in TRLLD. | 88.00% | 70.40% | 90.40% | 80.40% |
| The Hamilton Depression Rating Scale (HAM-D) is an appropriate tool for monitoring symptoms in TRLLD. | 88.00% | 70.40% | 90.40% | 80.40% |
| The Cornell Scale for Depression in Dementia is an appropriate tool for monitoring symptoms in TRLLD. * | 79.00% | 63.20% | 83.20% | 73.20% |
| **Category 2: Cognitive impairment, dementia, vascular depression** | | | | |
| Cognitive impairment can interfere with the accurate diagnosis of TRLLD. | 96.00% | 76.80% | 96.80% | 86.80% |
| Cognitive impairment can affect treatment response in LLD. | 100.00% | 80.00% | 100.00% | 90.00% |
| Cognitive screening should be routinely included in the TRLLD assessment. | 92.00% | 73.60% | 93.60% | 83.60% |
| Older adults with TRD, no prior history of depressive episodes, and dementia should not be included in the definition of TRLLD. They should be classified separately (e.g., as behavioural and psychological symptoms of dementia with treatment-resistant depressive symptoms). * | 75.00% | 60.00% | 80.00% | 70.00% |
| In patients with LLD, if apathy is the only remaining symptom after two or three adequate treatment trials, this does not indicate a TRLLD. * | 75.00% | 60.00% | 80.00% | 70.00% |
| **Category 3: Physical or mental comorbidities and TRLLD** | | | | |
| Certain conditions (e.g., thyroid disorders, testosterone deficiency, anaemia, vitamin deficiencies, etc.) should be screened for before diagnosing TRLLD. | 92.00% | 73.60% | 93.60% | 83.60% |
| If a patient has persistent depressive symptoms in the context of another primary psychiatric disorder despite receiving adequate treatment, this does not indicate TRLLD. * | 83.00% | 66.40% | 86.40% | 76.40% |
| Physical comorbidities (e.g., chronic pain or hypertension) should be adequately managed before confirming a diagnosis of TRLLD. * | 79.00% | 63.20% | 83.20% | 73.20% |
| **Category 4: Pharmacokinetics, pharmacodynamics, drug interactions and TRLLD** | | | | |
| Age-specific pharmacokinetic and pharmacodynamic characteristics should be considered when defining TRLLD. | 96.00% | 76.80% | 96.80% | 86.80% |
| **Category 5: Treatment adherence, tolerance and TRLLD** | | | | |
| Poor adherence is a common cause of treatment failure in older adults with depression. | 96.00% | 76.80% | 96.80% | 86.80% |
| In patients with Late-Life Depression (LLD), if one pharmacological treatment was discontinued due to side effects, it should not be considered a failed trial, even if depressive symptoms improved with this treatment. | 92.00% | 73.60% | 93.60% | 83.60% |
| The standard TRLLD assessment should include structured questions about adherence, with input from a proxy such as a partner or caregiver. * | 83.00% | 66.40% | 86.40% | 76.40% |
| Older adults with cognitive impairment require additional adherence monitoring before being classified as TRLLD. | 88.00% | 70.40% | 90.40% | 80.40% |
| **Category 6: Social, psychological factors and TRLLD** | | | | |
| Social isolation contributes to poor treatment outcomes in LLD. | 92.00% | 73.60% | 93.60% | 83.60% |
| Psychosocial stressors (e.g., financial difficulties, recent losses, or institutional living) should be routinely assessed before diagnosing TRLLD. * | 83.00% | 66.40% | 86.40% | 76.40% |
