# Supplementary material for: Definition of treatment-resistant late-life depression: Conclusions from a European Task Force Delphi process
Source: Eur Psychiatry. 2026 Jun 2;69(1):e68. doi: 10.1192/j.eurpsy.2026.12228 (PMC13359010; doi:10.1192/j.eurpsy.2026.12228)
Supplement: Pozuelo Moyano et al. supplementary material [file S0924933826122287sup001.zip › Supplementary_Table_S3_non_consensus_items_revisedproofs.docx]

**Supplementary Table S3. Sensitivity analysis of Round 2 agreement percentages under worst-case and best-case assumptions for non-responders: non-consensus items.**

Observed percentages are based on experts participating in Round 2 (n = 24). Worst-case and best-case percentages assume that all six non-responding experts disagreed or agreed, respectively, and are recalculated over the full panel (n = 30). The last column shows the average of the worst-case and best-case values. Items marked with † did not reach consensus in the observed 2nd SR results but would cross the 70% threshold under the best-case scenario.

|  | **Observed** | **Worst-case** | **Best-case** | **Average of the worst/best** |
| --- | --- | --- | --- | --- |
| Category 1: Global definition and clinical presentation Age definition & diagnostic criteria | | | | |
| Current adult TRD criteria are generally applicable to older adults. | 62.00% | 49.60% | 69.60% | 59.60% |
| Adults aged ≥85 years should be considered a distinct diagnostic subgroup (e.g., Very Late-Onset-TRD). | 58.00% | 46.40% | 66.40% | 56.40% |
| In recurrent depression, if a previously effective antidepressant no longer works, it should not count as an adequate trial for TRLLD; two or three different new antidepressants should be tried first. | 42.00% | 33.60% | 53.60% | 43.60% |
| Category 1: Global definition and clinical presentation Operational versus categorical definitions of TRLLD | | | | |
| If TRLLD is defined operationally, Stage I should be “failure of at least one adequate trial of a major antidepressant class.” † | 67.00% | 53.60% | 73.60% | 63.60% |
| Category 1: Global definition and clinical presentation Number of treatment failures & psychotherapy | | | | |
| TRLLD should be defined as occurring in patients who do not achieve response, defined as a 50% or greater reduction in symptom severity from baseline. † | 67.00% | 53.60% | 73.60% | 63.60% |
| In LLD, residual symptoms after two or three adequate treatment trials do not indicate TRLLD. † | 67.00% | 53.60% | 73.60% | 63.60% |
| Category 1: Global definition and clinical presentation Assessment tools | | | | |
| The Patient Health Questionnaire-9 (PHQ-9) is appropriate for monitoring symptoms in TRLLD. | 58.00% | 46.40% | 66.40% | 56.40% |
| The Geriatric Depression Scale (GDS) is appropriate for monitoring symptoms in TRLLD. | 62.00% | 49.60% | 69.60% | 59.60% |
| Category 2: Cognitive impairment, dementia, vascular depression | | | | |
| Older adults with TRD, no prior depressive history, and comorbid MCI should be classified separately (e.g., Organic syndrome with TRD symptoms), not included in TRLLD. | 42.00% | 33.60% | 53.60% | 43.60% |
| Older adults who meet criteria for vascular depression should be classified separately, not included in TRLLD. | 37.00% | 29.60% | 49.60% | 39.60% |
| Category 3: Physical or mental comorbidities and TRLLD | | | | |
| Chronic physical illnesses should be considered in the definition of TRLLD. † | 67.00% | 53.60% | 73.60% | 63.60% |
| Category 4: Pharmacokinetics, pharmacodynamics, drug interactions and TRLLD | | | | |
| When relevant, age-related pharmacokinetics and pharmacodynamics considerations should apply starting at age 60. | 62.00% | 49.60% | 69.60% | 59.60% |
| TRLLD should not be diagnosed without checking plasma levels when a co-medication induces hepatic enzymes affecting antidepressant metabolism. † | 67.00% | 53.60% | 73.60% | 63.60% |
| Medications with known depressogenic effects (e.g., beta-blockers) should be reduced or discontinued before labelling a case as TRLLD. | 58.00% | 46.40% | 66.40% | 56.40% |
| Category 5: Treatment adherence, tolerance and TRLLD | | | | |
| Adherence should be routinely assessed via plasma drug levels before diagnosing TRLLD. | 58.00% | 46.40% | 66.40% | 56.40% |
| Category 6: Social, psychological factors and TRLLD | | | | |
| Accessibility issues (e.g., access to ECT or blood level testing) should be considered in the definition of TRLLD. † | 67.00% | 53.60% | 73.60% | 63.60% |
